# A Feasibility Study of a Canadian Child Sexual Abuse Perpetration Prevention Program

**DOI:** 10.1177/10790632261460836

**Published:** 2026-06-18

**Authors:** Skye Stephens, Ian V. McPhail, Ainslie Heasman, Cory Gerritsen, Sarah Moss, Stéphanie Chouinard-Thivierge

**Affiliations:** 1 Department of Psychology, 3690 Saint Mary’s University, Halifax, NS, Canada; 2 Bloomberg School of Public Health, Johns Hopkins University, Baltimore, MD, USA; 3 7978 Centre for Addiction and Mental Health, Toronto, ON, Canada; 4 Department of Psychology, 33527 Thompson Rivers University, Kamloops, BC, Canada; 5 5622 Université de Montréal, Montreal, QC, Canada

**Keywords:** child sexual abuse, pedophilia, hebephilia, perpetration prevention, feasibility

## Abstract

Online or other child sexual exploitation or abuse (OCSEA) perpetration prevention programs are designed to provide services to individuals *prior to offending*. There is limited research on OCSEA perpetration prevention programs in jurisdictions with mandatory reporting. The present study is a mixed-method feasibility evaluation of the OCSEA perpetration prevention therapy program, *Talking for Change* (TFC). The feasibility study broadly addressed whether adult clients could be recruited to the program, the clinical profile of clients, and their satisfaction with the program. In total, there were 162 distinct individuals referred with the number of referrals increasing on an annual basis. Clients in the evaluation (*n* = 40) presented with risk relevant factors related to paraphilic interests and sexual self regulation problems with interpersonal challenges as well as several protective factors (e.g., empathy). The population had a high level of comorbid mental health problems, and many had engaged in prior CSEM use. Program satisfaction interviews resulted in five themes that highlighted the value of the group (e.g., promoting acceptance and accountability) and challenges (e.g., problems with a one size fits all approach). The results indicated that OCSEA perpetration prevention programs are feasible in mandatory reporting jurisdictions. Implications for program delivery are discussed.

Child sexual abuse is a preventable public health problem impacting approximately 9.5% of girls and 5.5% of boys worldwide (Piolanti et al., 2025). Over the past decade, there has been increased attention to the development of secondary perpetration prevention^1^ programs to reduce the public health burden of online or other child sexual exploitation or abuse (OCSEA) perpetration. OCSEA connotes forms of sexual exploitation against children that occur online (e.g., child sexual exploitation material [CSEM], online solicitation) and offline (e.g., child sexual abuse, commercial sexual exploitation of children). Secondary OCSEA perpetration prevention programs aim to support individuals at-risk of engaging in OCSEA. Although these programs are optimally accessed *prior to offending,* another potential target group that often present for services includes individuals engaging in OCSEA who have not been detected by the legal system (Beier et al., 2009). The present study provides feasibility results for the first federally funded secondary OCSEA perpetration prevention program in Canada, *Talking for Change* (TFC), with a focus on the psychotherapy program.

## Secondary OCSEA Perpetration Prevention Program Target Group

Prior to discussing the research on existing secondary OCSEA perpetration prevention programs (herein referred to as perpetration prevention programs) that offer psychotherapy, it is important to discuss the target group for these programs. Most perpetration prevention programs that offer psychotherapy have focused on service provision to those with sexual interest in prepubescent and/or pubescent children (Beggs Christofferson et al., 2025; Beier, 2021; Stephens et al., 2022). The decision to focus on this target group is understandable when one considers that sexual interest in children is a strong factor associated with both the onset and persistence of sexual offending (McPhail et al., 2019) and onset typically occurs during adolescence making the individual aware of this prior to offending behavior (Seto, 2018). Thus, individuals would be aware of this interest early on and could engage with perpetration prevention programming prior to offending.

While sexual interest in children is a central risk factor for OCSEA perpetration (e.g., McPhail et al., 2019), there is a broad range of psychosocial risk factors that are conceptually and empirically linked to the onset and persistence of OCSEA perpetration. Several meta-analytic reviews suggest that cognitive distortions relating to children and sex (Helmus et al., 2012), general and sexual self-regulation problems (Hanson & Morton-Bourgon, 2005), strong attachments to children and childhood (McPhail et al., 2013), intimacy deficits (Whitaker et al., 2008), and antisociality (Hanson & Morton-Bourgon, 2005) are empirically supported risk factors for OCSEA perpetration. It has been argued that programs should offer services to a broader target group beyond just those with sexual interest in children (Stephens et al., 2022).

## Existing Perpetration Prevention Programs

There has been increased interest in perpetration prevention programs with many examples of programs offered across different modalities (e.g., helplines, self-guided interventions; Stephens et al., 2022; Seto et al., 2024). Traditionally, these programs have often focused on adults with fewer programs focused on adolescents (Seto et al., 2024); however, this has been rapidly changing with the development of several adolescent specific programs (e.g., Help Wanted; Shields et al., 2020; What’s Ok; Stop it Now!, 2026). Given that the current feasibility evaluation focuses on a perpetration prevention psychotherapy program for adults, the below discussion focuses explicitly on these types of programs due to space constraints.

In the domain of traditional psychotherapy programs, the Dunkelfeld project in Germany was one of the first to provide perpetration prevention services. The program aimed to prevent OCSEA among those with sexual interest in children who were not involved in the legal system via the provision of comprehensive assessment and therapeutic services (Beier et al., 2009). A major focus of the program is on helping individuals understand and manage personally relevant dynamic risk factors for OCSEA perpetration (e.g., cognitions that support sexual offending, intimacy deficits, Beier, 2021). The program has been expanded to include youth aged 12–18 (Beier et al., 2016).

Beier et al. (2015) conducted a non-randomized waitlist-controlled study of the adult-focused Dunkelfeld program. They found several significant changes on a range of risk-relevant characteristics (e.g., offense supportive attitudes) in the treatment group; however, there was no significant reduction in self-reported online (i.e., CSEM) or offline (i.e., contact) offending. Mokros and Banse (2019) re-analyzed the Dunkelfeld study results from the Beier et al. (2015) original study to directly compare the treatment and control group on treatment change (Beier et al. did not include this comparison). There was no significant change in the treatment group compared with the waitlist control group; however, the average effect size suggested a small effect (Mokros & Banse, 2019).

More recently, Beier et al. (2024) provided pre-post data on 56 completers of the Dunkelfeld program (pre, post, and follow-up which ranged from 1 to 11 years). Of those who completed the program, the use of CSEM decreased from pre to post treatment (from 82.1% to 62.5%) but slightly increased during the follow-up period (to 75% of the sample) though it remained lower than pre-treatment levels. While individuals persisted in CSEM use, a notable finding was that the severity of the CSEM material decreased over time. The results suggested that both the frequency and severity of CSEM use decreased throughout treatment. Of the individuals who committed contact offenses against children (46.4%), two sexually reoffended with a contact offense. There were reductions in cognitive victim empathy deficits and offense-supportive attitudes across time; however, these changes were not maintained to the same extent during the follow-up period underscoring the need for ongoing care. Although longer-term follow-up data is valuable, there was no control group that allowed comparison between those who did and did not complete the program.

Another example of an OCSEA perpetration prevention program is *Prevent It!*, which is a self-guided online intervention for those currently accessing CSEM (Lätth et al., 2022). Thus, the target group of this program is different than the Dunkelfeld in that it is not just limited to those with sexual interest in children because it includes individuals engaging in online sexual offending, many of whom would be unknown to legal authorities (thus in some ways it is more closely related to a tertiary prevention program). Clients in the program completed eight weeks of internet-based cognitive behavioral therapy (CBT) with modules focused on risk management, sexual self-regulation strategies, and restructuring cognitions that support or condone CSEM use. Lätth et al. provided promising results on the efficacy of the program and found that compared with an active placebo condition, those in Prevent It! program demonstrated a significant decrease in CSEM use compared with the control group (*d* = 0.18 from pre-to post-treatment). They also found that change was maintained over a one-month follow-up period (*d* = 0.20).

As noted, existing perpetration prevention programs focus on helping clients manage and reduce risk factors for onset and persistence of offending; however, it is clear from existing research that individuals who access these interventions or have a sexual interest in children also experience a broad range of clinically significant needs, and in some instances, may be engaging in OCSEA undetected by the legal system. These populations tend to report high rates of anxiety and depressive symptomatology (Gerwinn et al., 2018), stigma-related stress (see Cantor & McPhail, 2016; McPhail, 2024 for reviews), and suicidality (Elchuk et al., 2022; Ingram et al., 2023). Individuals with sexual interest in children also report elevated rates of sexual abuse victimization in their own childhood (see Cantor & McPhail, 2016; McPhail, 2024 for reviews). When putative members of the client population are asked what they would want mental health professionals to help them with in treatment, these individuals tend to emphasize more general mental health concerns over risk-relevant treatment targets (Stephens et al., 2026).

Taken together, the available evidence raises questions about whether it is feasible for perpetration prevention programs (e.g., TFC, Dunkelfeld) to provide adequate coverage of the clinical needs of the target population (i.e., dynamic and acute risk factors for OCSEA perpetration, mental health concerns, substance abuse, stigma-related stress, shame, and ongoing impacts of childhood sexual abuse). One substantive limitation to the evidence suggesting a broader range of clinical needs in this population is that the findings are often from samples recruited from online forums. At present, data on this broader range of clinical needs (e.g., mental health problems such as depression and anxiety, substance use, overall functional impairment) are generally unavailable for help-seeking individuals who self-refer to perpetration prevention programs for support, as existing program evaluations typically focus on risk-relevant needs (cf., Beier et al., 2015; Lätth, 2022).

## OCSEA Perpetration Prevention Programs and Mandatory Reporting

When interpreting past findings, it is important to consider that the Dunkelfeld program emerged in a jurisdiction where mental health professionals are not required to report situations where children are at-risk of or are being sexually abused (Beier et al., 2009, 2015) or in contexts where services are provided anonymously (Prevent It; Lätth et al., 2022). At present, there is no published data on whether OCSEA perpetration prevention programs are feasible within mandatory reporting jurisdictions where clinicians are mandated reporters (e.g., Canada; McPhail et al., 2018). Mandatory reporting presents plausible critical issues related to client recruitment to perpetration prevention programs and retaining them within ongoing psychotherapy (Beggs Christofferson et al., 2025).

The issue of mandatory reporting requirements, and its impacts on referral rates, is of paramount consideration in Canada where child protection legislation mandates the reporting of suspected child abuse in all Canadian provinces, including if there is evidence to suggest that a child is at-risk of being abused (McPhail et al., 2018). “At-risk” is poorly defined within legislation and is open to interpretation which can lead to uncertainty in clinical decision making when a client presents with a sexual interest in children (e.g., Stephens et al., 2022). Further, mandatory reporting requirements are an identified barrier for clients who may be interested in receiving support from perpetration prevention programs (Levenson et al., 2020; Levenson & Grady, 2019; McPhail et al., 2018; Stephens et al., 2022; Swaby & Lievesley, 2022). At present, it remains an open question whether it is possible and feasible to recruit individuals into a secondary OCSEA perpetration prevention program in a mandatory reporting jurisdiction. It is plausible that perpetration prevention programs in mandatory reporting jurisdictions would receive few referrals, given the help-seeking barriers that clients have identified related to these reporting requirements (Chronos et al., 2024).

## Present Study

The present study is a mixed-method feasibility evaluation of the OCSEA perpetration prevention therapy program, *Talking for Change* (TFC). The TFC program was launched in 2021 as the first federally funded perpetration prevention program in Canada. One of the aims of TFC is to provide non-anonymous therapy to adults with a sexual interest in children and/or those concerned about their risk of OCSEA perpetration, which could include individuals who have engaged in OCSEA but are unknown to legal authorities (see https://talkingforchange.ca for more information).

A feasibility evaluation is the first step in a rigorous evaluation of an intervention and focuses on process issues and informs whether an effectiveness evaluation can be conducted (e.g., Shanyinde et al., 2011; Tickle-Degnen, 2013). The feasibility evaluation is a natural starting point because it identifies the components of a program that work well and identifies components that need to be adjusted before investing more significantly in a program.

The present feasibility evaluation addressed three research objectives. *Research objective 1* (RO1) addressed whether individuals could be recruited to TFC via an examination of the flow of client referrals. *Research objective 2* (RO2) addressed the characteristics and needs of individuals (mental health needs and risk-relevant factors) seeking TFC to build a clinical profile of the sample. *Research objective 3* (RO3) addressed the perceptions of and satisfaction with the TFC program among clients.

The present study is novel as it is the first published study of an OCSEA perpetration prevention program that is a traditional psychotherapy program offered in a mandatory reporting jurisdiction. To our knowledge, it is the first study to address client satisfaction with these services and incorporate qualitative data, which is important given disagreement amongst stakeholders as to what should be covered in such programs (Stephens et al., 2026). These findings have implications for program development and evaluation in jurisdictions with similar reporting laws (e.g., United States). Lastly, the results are important in the context of modifying the TFC program and informing more rigorous research focused on program efficacy, which is necessary to establish that TFC is effective for this client population.

## Method

The authors take responsibility for the integrity of the data, the accuracy of the data analyses, and have made every effort to avoid inflating statistically significant results.

### Participants

The present study is based on 162 distinct individuals (adults) referred to the program from August 2021 to September 2024 across several Canadian provinces. The program was offered at a large mental health hospital and in a private practice setting. Individuals were eligible for psychotherapy services within the TFC program if they were 18 years of age or older and fit the target population (i.e., they either had sexual interest in children and/or were concerned about their risk of OCSEA perpetration). Individuals were ineligible if they had a severe mental illness that was untreated or in an acute phase, were mandated to participate in treatment by the criminal legal system following a charge/conviction for a sexual offense, had a current charge for a sexual offense, or had involvement with the criminal justice system for a sexually motivated offense within the last ten years. Approval for the evaluation was granted through the Quality Project Ethics Review at the hospital and a Research Ethics Board at a local university.

Table 1 provides basic demographic information for those referred to the program. Clients ranged in age from 18 to 67 with an average age of 31.4 (*SD* = 11.3). Clients were mostly male (88.7%), White (71.2%), single (71.2%), and had some form of postsecondary education (60.0%). Despite a high level of education, a significant number of clients were unemployed (27.4%) or on some form of social assistance (23.5%).

**Table 1.** Demographic and Offense History Characteristics of Those Referred to Talking for Change (n = 162)

​	*M* (*SD*) [Range]	% (*n*/*N*)
Age (*n* = 105)	31.4 (11.3) [18–67]	
Gender identity		
Man		88.7 (94/106)
Woman		4.7 (5/106)
Non-binary		1.8 (2/106)
Trans		2.8 (3/106)
Other		1.8 (2/106)
Prefer not to say/Unknown		34.6 (56/162)
Ethnicity		
White		71.2 (74/104)
First Nations		0.9 (1/104)
Black		4.8 (5/104)
Asian		12.5 (13/104)
Latin American		0.9 (1/104)
Middle East		4.8 (5/104)
Mixed heritage		4.8 (5/104)
Unknown		35.8 (58/162)
Marital status		
Single		71.2 (74/104)
Dating		5.8 (6/104)
Committed relationship		8.6 (9/104)
Married/Common-law		11.5 (12/104)
Divorced/Separated		2.9 (3/104)
Unknown		35.8 (58/162)
Highest level of education		
Less than high school		10.0 (10/100)
High school/GED		30.0 (30/100)
College/University		60.0 (60/100)
Unknown		38.3 (62/162)
Employment status		
Employed full-time		34.3 (35/102)
Employed part-time		11.8 (12/102)
Social assistance		23.5 (24/102)
Retired		2.0 (2/102)
Self-employed		1.0 (1/102)
Unemployed		27.4 (28/102)
Unknown		37.0 (60/162)
History of sex offense charges		
Yes		7.7 (12/155)
Contact sexual offense		1.3 (2/155)
CSEM offense		2.6 (4/155)
Unknown		3.9 (6/155)
No		87.7 (136/155)
Unknown		4.3 (7/162)
History of sex offense convictions		
Yes		2.7 (4/148)
Contact sexual offense		0.0 (0/148)
CSEM offense		2.0 (3/148)
Unknown		0.7 (1/148)
No		88.5 (131/148)
Unknown		8.6 (14/162)

*Note.* The information in this table was gathered from program administrative data from the self-referral form that individuals completed and the subsequent screening process.

### The Talking for Change Psychotherapy Program

The TFC clinical service offers non-anonymous group-based or individual psychotherapy to adults. Services are primarily offered in a virtual modality with the option for in-person assessment and therapy in some circumstances. The initial psychotherapy offering was 12 sessions but later expanded to 16 sessions after the first group to provide more time to cover key content. The psychotherapy group sessions were conducted virtually and facilitated by registered mental health professionals, or with their trainees who were often clinical psychology residents; the facilitators always included an experienced, doctoral-level psychologist that was part of the development team for the program. Clients referred to the TFC psychotherapy program underwent several assessment and survey procedures (see Measures and Procedures sections for details).

The core focus of the TFC psychotherapy program involves providing clients with an understanding of the psychosocial factors that are associated with OCSEA perpetration (i.e., dynamic risk factors), an understanding of the risk factors that may be present in their lives, training in the use of psychological skills that will help them manage risk factors and potentially risky situations in the future, and ways to continue to move towards living fulfilling and offense-free lives. The program also includes skills directly relevant to facilitating improvement in wellbeing. This overarching orientation aligns with TFC as a perpetration prevention program, which aims to help individuals avoid future offending. Treatment programs that intend to prevent offending are informed by general, evidence-based rehabilitative frameworks that indicate such programs are most effective when interventions are used to specifically address and ameliorate known risk factors for offending (Bonta & Andrews, 2023).

The TFC psychotherapy program focuses on a range of clinical issues. Early sessions focus on motivation and goals for treatment, personal values, psychoeducation about sexual interest in children (e.g., pedophilia and hebephilia), and self-compassion-focused interventions. Later sessions in the program build on this foundational content and provide interventions to clients to help them build psychological skills like mindfulness, emotion regulation, cognitive restructuring, understanding and managing sexual fantasies and arousal, interpersonal communication, and managing high risk situations. The TFC psychotherapy program is integrative in orientation, drawing interventions from cognitive behavioral, acceptance and commitment, dialectical behavioral, and compassion-focused approaches to therapy.

### Measures

There were a wide range of formal measures administered at various time points throughout the program. For the purposes of analyses, information was extracted from either program administrative or clinical files (for evaluation clients only; see below). Several self-report questionnaires were administered at the time of the diagnostic assessment as part of a standard battery that is a component of routine clinical practice for all clients seeking support for their sexual behavior at the hospital (this process was matched in the private practice for consistency). The purpose of the self-report battery was to provide an assessment of a range of clinical concerns focused on mental health needs and functional impairment. Additionally, risk and protective factor assessment interviews were conducted after the diagnostic interview if the client was eligible for TFC and consented to the evaluation to identify relevant risk-related needs and protective factors. These measures are all outlined below.

#### Questionnaires: Self-Report Mental Health Concerns and Functional Impairment

The World Health Organization Disability Assessment Schedule 2.0 (WHODAS 2.0; Üstün et al., 2010) is a self-report measure assessing the impact of physical/mental health problems on daily functioning (i.e., disability). The brief screening 12-item version was used in the present study. For each item, participants had to indicate the level of impairment over the past 30 days, from one (i.e., none) to five (i.e., extreme or cannot do). Total scores range from 12 to 60 and higher scores indicate greater functional impairment. Levels of functional impairment/disability were categorized into no impairment (scores from 0 to 12), mild (scores from 13 to 24), moderate (scores from 25 to 36), severe (scores from 37 to 48), and extreme impairment (scores from 49 to 60). Psychometric evaluations of the WHODAS 2.0 have demonstrated acceptable internal consistency, good test-retest reliability, and validity across clinical and general populations, and its utility as a comprehensive measure of disability in research and clinical settings has been confirmed (Federici et al., 2017; Gondim et al., 2025; Üstün et al., 2010).

The Compulsive Sexual Behavior Disorder – Diagnostic Inventory (CBSD-DI formerly referred to as HD-CBSD; Reid et al., 2019) is a nine-item self-report measure assessing symptoms and consequences of compulsive sexual behavior that correspond to the diagnostic criteria in the International Classification of Diseases. For each item, participants responded to what extent the statement corresponds to their situation (i.e., “this has never been true” = 0 or “this has been true in my lifetime but not during the last 12 months” = 1; and “this has been true for at least 6 months during the last 12 months” = 2). The possible total scores range from 0 to 18. Higher total scores are indicative of higher levels of impairment and distress due to compulsive sexual behavior. Psychometric evaluations have shown the measure has adequate internal consistency, test-retest reliability, and convergent validity (Grubbs et al., 2023).

The Patient Health Questionnaire (PHQ-9; Kroenke et al., 2001) is a nine-item self-report measure assessing depressive symptom severity. Participants rated how often nine depressive symptoms have been bothering them over the past two weeks, using a scale from 0 (i.e., “not at all”) to 3 (i.e., “nearly every day”). Possible total scores range from 0 to 27, and higher total scores indicate greater levels of depression severity. Total scores on the PHQ-9 were used to identify clients with increasing severity of depressive symptoms, which include minimal or no depressive symptoms (scores from 0 to 4, no treatment needed), mild depressive symptoms (scores from 5 to 9, consider treatment based on duration of symptoms and impairment), moderate depressive symptoms (scores from 10 to 14, symptoms expected to cause some impairment and treatment is needed), moderately severe depressive symptoms (scores from 15 to 19, symptoms are pronounced and result in functional impairment suggesting treatment is required), and severe depressive symptoms (scores from 20 to 27, significant symptoms that impact daily life and need for immediate treatment). Numerous studies have evaluated the PHQ-9, and demonstrated adequate construct validity, test-retest reliability, and internal consistency of this scale in measuring depression with various populations in a clinical setting, as well as with community populations (e.g., Feng et al., 2016; Patrick & Connick, 2019).

The Generalized Anxiety Disorder-7 (GAD-7; Spitzer et al., 2006) is a seven-item self-report measure assessing severity of anxiety symptoms. Participants were asked to rate to what extent the seven symptoms have been bothering them over the past two weeks, using a scale from 0 (i.e., “not at all”) to 3 (i.e., “nearly every day”). Possible total scores range from 0 to 21, with higher total scores indicating greater levels of anxiety symptoms severity. Total scores on the GAD-7 were used here to identify clients with increasing severity of anxiety symptoms, which include minimal anxiety symptoms (scores from 0 to 4, anxiety symptoms are minimal/not present and not suggestive of diagnosis), mild anxiety symptoms (scores from 5 to 9, symptoms are noticeable but can be managed), moderate anxiety symptoms (scores from 10 to 14, anxiety symptoms are more significant and impairing), and severe anxiety symptoms (scores 15 and higher, significant anxiety symptoms that are consistent with an anxiety disorder). The psychometric properties of the GAD-7 have been widely examined among different clinical populations and results indicate acceptable convergent and discriminant validity, internal consistency, and test-retest reliability (e.g., Johnson et al., 2019; Löwe et al., 2008).

The Alcohol Use Disorders Identification Test (AUDIT; Saunders et al., 1993) is a ten-item self-report measure assessing drinking behaviors and alcohol consumption. The AUDIT contains questions regarding different domains, namely alcohol consumption, drinking behavior and dependence, and alcohol-related harm. For each question, participants responded on a scale from 0 (i.e., “never”) to 4 (i.e., “daily or almost daily”). Possible total scores range from 0 to 40, with higher total scores indicative of greater levels of alcohol-related risks. Total scores on the AUDIT identified clients with increasing alcohol-related problems, which include absence of problems (scores of 0), low-risk consumption (scores from 1 to 7), hazardous alcohol consumption (scores from 8 to 15), and consumption indicative of possible alcohol dependence (scores of 15 or more). Each category on the AUDIT is tied to specific recommendations for action (e.g., for hazardous consumption, the recommended approach involves motivational approaches). The AUDIT has demonstrated strong psychometric properties, with high internal consistency, good test-retest reliability, and validated use across diverse populations in different settings (e.g., Reinert & Allen, 2007).

The Drug Abuse Screening Test (DAST-20; Skinner, 1982) is a self-report measure used to assess problems with drug abuse. Participants first asked to indicate if they had used drugs in the past 12 months and, if this item was endorsed, they were then asked to complete 20 dichotomous “Yes” or “No” items pertaining to their drug use over the past 12 months. Possible total scores range from 0 to 20, with higher scores indicating greater severity of drug problems. Total scores on the DAST-20 were used to identify clients with different severity of problems with drug use, which include absence or minimal problems with drug use (score of 0), low severity of drug use (scores from 1 to 5), intermediate severity of drug use (scores from 6 to 10), substantial problems with drug use (scores from 11 to 15), and severe problems with drug use (scores from 16 to 20). A total score above 11 suggests the need for treatment or intensive intervention. Like the AUDIT, these ranges on the DAST-20 are tied to recommended actions (e.g., intermediate severity suggests need for outpatient treatment). The DAST-20 has been extensively evaluated for its psychometric properties across various populations and settings, and the results generally suggest the acceptable reliability, internal consistency, as well as convergent and discriminant validity of this scale (e.g., Johnson et al., 2024; Villalobos-Gallegos et al., 2015).

#### Interview-Based Measures of Clinical Needs

In addition to the self-report questionnaires described above, a comprehensive clinical interview was the cornerstone of the diagnostic assessment. The diagnostic assessment was conducted by a registered healthcare professional (or their trainee) if clients were deemed eligible for TFC after an initial screening call. The diagnostic assessment was focused on a psychosexual assessment, mental health functioning, and a brief psychosocial history. The purpose of the diagnostic assessment was to determine TFC psychotherapy eligibility and to provide referrals for clinical and other services outside of TFC, as needed.

A coding form was developed for the purposes of the evaluation to extract information from the client’s referral and diagnostic assessment report maintained in electronic medical records. Information extracted included past mental health diagnoses (as reported by the client on their referral form or from a physician in the small number of cases where the client was directly referred by a physician); current mental health (diagnoses, inclusive of paraphilic disorders, rendered during the diagnostic assessment); past and current treatment for mental health and paraphilic disorders reported by the client (inclusive of psychotropic medications, psychosocial interventions, and past sexuality-specific interventions); suicidality (past and current self-reported suicidal ideation and a history of suicide attempts); self-reported adverse childhood experiences (any abuse, emotional abuse, physical abuse, sexual abuse, neglect, witness domestic violence; other forms of abuse/adversity), sexological history (including paraphilic interests that were present and may or may not have met the threshold for a disorder). Sexual offense history was also canvassed via client self-report and was inclusive of self-reported detected and undetected contact sexual offending against children, non-contact offending, and CSEM history.

In addition to the diagnostic assessment, evaluation clients subsequently participated in an interview that involved an assessment of risk and protective factors with a research staff member prior to starting the program. The Violence Risk Scale: Sex Offense version (VRS:SO; Olver et al., 2007) is an interview-based measure of risk factors for sexual offending. The present study focused on the 17 dynamic risk factors which load onto the following three factors: sexual deviance (e.g., items assessing sexually deviant lifestyle, sexual compulsivity, risk-relevant sexual preferences); general criminality (e.g., items assessing psychopathy, aggression, impulsivity); and treatment responsivity (e.g., items assessing insight, compliance with treatment; Olver et al., 2022). Each VRS:SO item is scored by an observer on a four-point scale (0 to 3) with a score of 2 or 3 indicating a significant connection between a risk factor and sexual offense risk, which means that it is an identified treatment need for a given client. The total dynamic score, the three factors (sexual deviance, criminality, and treatment responsivity) and individual scores (both the individual score and the percentage for which the factor was present at a two or three signifying a treatment need) are reported.

The VRS:SO was developed and validated on individuals with a history of detected sexual offending, which is a tertiary prevention client population. Given that TFC was developed to offer services to individuals who either have not offended or do not have a history of detected offending, the VRS:SO was used “off-label” in the present study. We did this because no risk assessment measures exist for use with perpetration prevention clients. In addition, the off-label use of the VRS:SO provided an understanding of potential treatment needs for this population, helped evaluate the feasibility of using risk assessment instruments in this population, and helped identify shortcomings of existing measures. Finally, the empirical evidence published to date tends to support the same risk factors found in individuals with detected sexual offense histories and those who are in the client populations served by secondary prevention programs (for review, see McPhail, 2024). The VRS:SO has strong support for a three-factor structure and for its convergent, divergent, and predictive validity (Olver et al., 2022). Further, client improvement over the course of treatment on the VRS:SO dynamic risk factors has been linked to reductions in sexual recidivism (Olver et al., 2022; Olver & Stockdale, 2020). In the present sample, interrater reliability for the VRS:SO—across seven clients—was excellent (*ICC* = .93).

The Structured Assessment of Protective Factors against Sexual Offending (SAPROF-SO; Willis et al., 2021) was used to assess for protective factors that mitigate risk for sexual offending. The SAPROF-SO contains 14 protective factors that assess an individual’s recent functioning (generally over six months, but in some instances items can be rated over 12 or 24 months) across three broad domains of resilience (i.e., internal capacity to cope with environmental stressors that are relatively stable), adaptive sexuality (i.e., prosocial and adaptive sexual interests and behavior, management of atypical interests), and prosocial connection and reward (i.e., adaptive social connections and engagement in prosocial activities). Each item is scored on a scale from 0 to 4 and is rated by an observer using information from interviews and/or clinical records, with scores of 0 indicating the absence of a protective factor and scores of 4 indicating a high level of protection. For the SAPROF-SO, the total score, domain scores, and individual scores are provided. A protective factor was deemed to be present if a score of 2 to 4 was assigned, which corresponds to the protective factor being present to some extent or to the full extent.

The SAPROF-SO has a small body of research that suggests it has high interrater reliability, good construct validity, and predictive validity (e.g., Nolan et al., 2022; Willis et al., 2021). As with the VRS:SO, the SAPROF-SO is meant to be used with those who have a history of detected sexual offending, we used it off-label to gain a better sense of protective factors and strengths in this client population. Interrater reliability for the SAPROF-SO—across 10 clients—was excellent (*ICC* = .94).

### Program Satisfaction Interview

A semi-structured program satisfaction interview was administered by the research analyst after the completion of the TFC psychotherapy program. The research analyst conducting the interviews was not involved in the delivery of the TFC psychotherapy program. The purpose of the satisfaction interview was to better understand the experiences of clients in the program and to identify areas of the program that needed modification. Interview questions included asking clients about their experience with various aspects of the program (e.g., referral, assessments), areas that could be improved, and their experience in the group (e.g., transformative moments in group). See the Online Supplement for the full interview guide.

### Procedure

**Figure 1.**
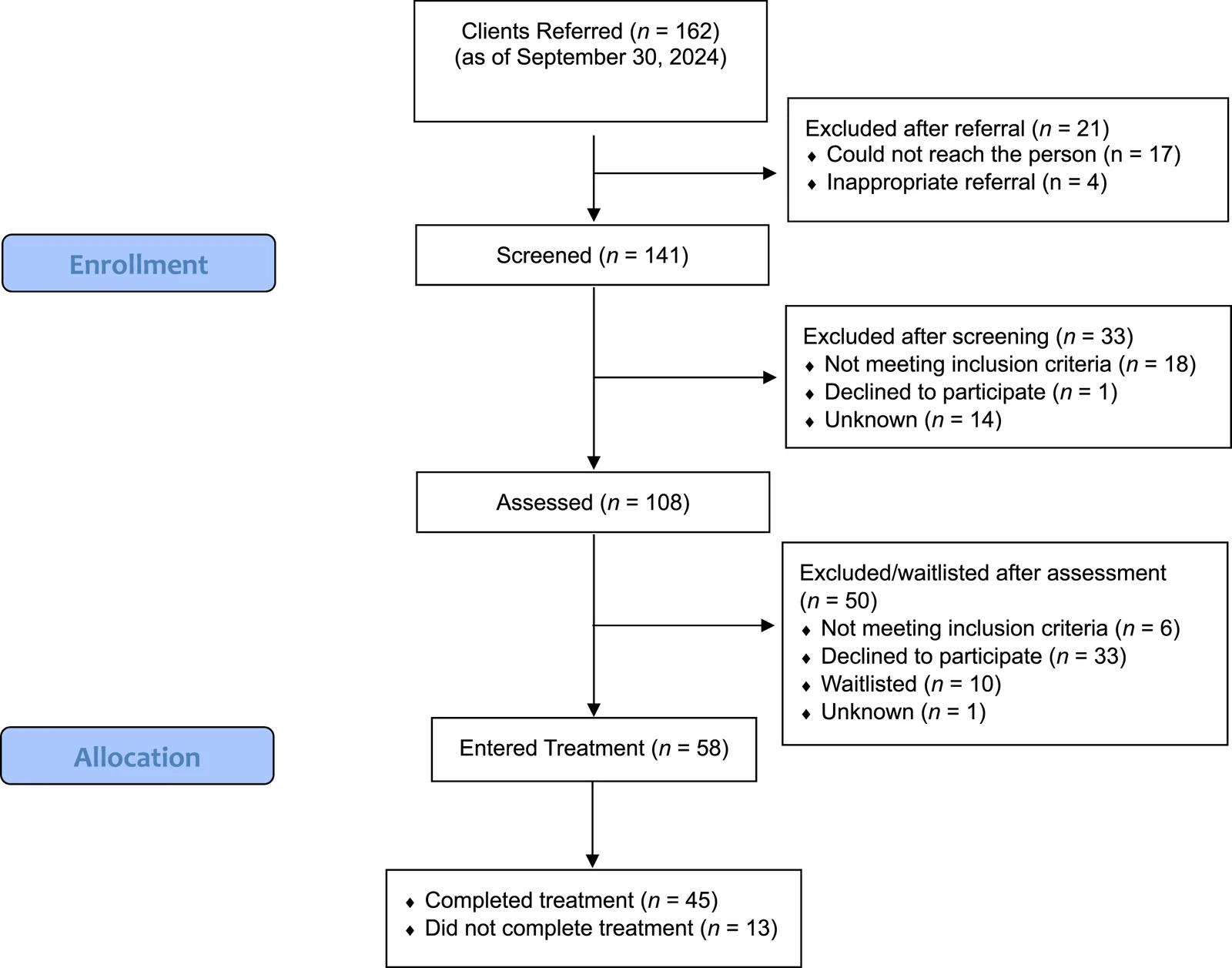
Flow chart of Total Number of TFC Clients Referrals

Figure 1 provides a flowchart illustrating how the 162 distinct adults referred to the program progressed through the clinical services offered by TFC (see RO1 results section for additional discussion of the Figure). Clients were referred via self-referral (*n* = 104), a referral from a physician internal to the hospital housing the TFC program (*n* = 22), or a referral from a physician external to the hospital (*n* = 36). In the private practice setting, only self-referrals were possible. Following receipt of a referral, TFC clinical staff conducted a screening call to provide a brief overview of the program, discuss limits to confidentiality, and ensure that clients did not have current or recent legal involvement or other concerns which would have deemed them ineligible for the program. There were 21 clients excluded after referral but prior to receiving a screening call (minimum of two attempts were made) with the majority (*n* = 17) excluded because they did not meet inclusion criteria, such as having current legal charges. There were 141 clients who proceeded to the screening call and an additional 33 clients were excluded (e.g., 18 did not meet inclusion criteria due to legal involvement).

Clients that appeared eligible based on the screening call (*n* = 108) were then assessed (herein referred to as the diagnostic assessment) by a TFC clinician (see above), which took 2 to 3 hours to complete. The diagnostic assessment included a formal interview and completion of clinic questionnaires (WHODAS 2.0, CSBD-DI, GAD-7, PHQ-9, AUDIT, DAST-20)^2^. As part of the diagnostic assessment, clients were informed of treatment recommendations (inclusive of TFC eligibility) and given diagnostic feedback. Clients offered TFC therapy at the hospital were able to attend the program in a virtual, group therapy format. If group therapy was deemed inappropriate for a given client, individual psychotherapy was offered (e.g., client was assigned female at birth, neurodiverse clients who required adapted treatment content or delivery to meet their specific needs). Clients offered TFC therapy in the private practice setting were offered virtual individual psychotherapy due to client numbers.

Following participation in a diagnostic assessment, 58 clients were eligible for TFC. In total, 45 clients completed treatment and 13 did not (9 of these 13 clients dropped out and the remaining four were removed). Clients who agreed to participate in the TFC psychotherapy program were offered the opportunity to participate in additional evaluation procedures being conducted by program staff. Clients who were interested in participating in the additional evaluation procedures met separately with a research analyst who provided an overview of the procedures and sought their consent to participate. The research analyst was not involved in the clinical administration of the program. Clients who consented to participate in the additional evaluation procedures completed another appointment in which dynamic risk (via the VRS:SO) and protective factors (via the SAPROF-SO) were assessed (see above).^3^ At the conclusion of the program, clients who consented to participate in the additional evaluation procedures were given the opportunity to participate in a 60-minute virtual program satisfaction interview. All interviews were audio-recorded, and the live transcription feature on the virtual platform was used to create an initial transcript of the interview. After the interview, interviews were transcribed, de-identified by the research analyst, and cross-checked against the audio recording for accuracy.

### Data Analysis

RO1 focused on whether clients could be recruited to the TFC program. To do so, we relied on broader program data maintained by the clinic that related to the number of distinct adults referred to the program (*n* = 162). RO2 focused on the characteristics and needs of individuals to better understand the client profile of the sample. To accomplish this objective, program and evaluation data are used to provide information about mental health symptoms, suicidality, and adverse childhood experiences via information gathered during the diagnostic interview or via self-report questionnaires. Further, risk and protective factors are available for clients who participated in additional evaluation procedures (*n* = 40).

RO3 addressed client satisfaction with the TFC psychotherapy program for those who completed the evaluation and the group treatment program prior to program modifications based on this feedback (*n* = 12). Clients who engaged in individual therapy were offered a program satisfaction interview but were excluded from the analyses because the program differed in that clients were typically screened into individual therapy based on clinical considerations and received an adapted version of the TFC program.

Thematic analysis, a qualitative approach used to identify themes and patterns in data relevant to the research question (Braun & Clarke, 2006), was used to analyze interview data. Thematic analysis is a flexible and diverse approach that recognizes that the researcher’s perspective is important as researchers are actively involved in knowledge production via the generation of themes (Braun & Clarke, 2006, 2021). A single coder (first author) with experience in qualitative research completed the coding and qualitative analysis but sought feedback from team members, including the research analyst who conducted the interviews. From a reflexivity perspective, the first author is a clinical-forensic psychologist with clinical and research expertise in the area of perpetration prevention programming and sexual offending against children. Most critically, they assisted in the co-development of the group but were *not* responsible for conducting therapy with those who participated in the program satisfaction interviews.

Braun and Clarke’s (2006, 2021) iterative six-stage process for TA was broadly followed. The first step involved an initial process of data familiarization via review of the transcripts; however, there was a slight deviation from the TA process in that the first author created a preliminary codebook to help guide coding and increase transparency. The codebook was frequently revised throughout the analytic process when new codes appeared to be useful and previously coded interviews were re-reviewed to ensure the appropriateness of coding. Codes were generated using an inductive approach and based on the semantic context. At the conclusion of coding the interviews, the codes were reviewed and organized into provisional themes. After the provisional themes were created, the first author reviewed the content for each theme and cross-checked against the interview data to ensure they were coherent. Feedback from the research team was also incorporated to help ensure credibility.

## Results

### RO1: Referrals to TFC

Figure 1 provides an overview of the number of clients referred to the TFC program across both sites. Between August of 2021 and September of 2024, 162 individuals were referred to the TFC program. Of the 162 individuals referred, 141 were screened, and 108 completed the diagnostic assessment. A total of 58 clients entered treatment (35.8%) and 45 subsequently completed treatment (77.6% of those eligible for treatment and 27.8% of the total sample). Few individuals in the sample had a self-disclosed official history of sexual offending (investigation, arrests, charges, or convictions). In total, 7.7% (*n* = 12) reported a historical sexual offense charge and 2.7% (*n* = 4) reported a conviction for a sexual offense (see Table 1 for other demographic characteristics). Using broader program data, the TFC program saw year-on-year increases in referrals, with 29 referrals in 2021 (monthly referral rate = 2.4), 45 referrals in 2022 (monthly referral rate = 3.8), 56 referrals in 2023 (monthly referral rate = 4.7), and 73 referrals in the first nine months of 2024 (monthly referral rate = 8.1).

### R02: Clinical Profile of TFC Clients

Table 2 provides an overview of mental health disorder diagnoses. Among the 162 individuals referred, 51.9% (*n* = 84) reported a past mental health disorder diagnosis, 11.1% (*n* = 18) reported no past diagnosis, and for 37% *(n* = 60) this information was unknown. In terms of current diagnoses for the 108 clients who underwent the assessment, the most frequent past mental health disorder diagnoses were Depressive Disorder (32.4%), Attention Deficit Hyperactivity Disorder (20.3%), and Anxiety Disorders (17.6%). For paraphilic disorders, a small minority of TFC clients reported a previous paraphilic disorder diagnosis (2.8%).

**Table 2.** Past and Current Mental Health Diagnoses of Talking for Change (TFC) (*n* = 108)

​	Past Diagnosis % (*n*)	Diagnosed in TFC % (*n*)
Anxiety disorder	17.6 (19)	0.9 (1)
Depressive disorder	32.4 (35)	3.7 (4)
Substance use disorder	9.2 (10)	1.8 (2)
Post-traumatic stress disorder	1.8 (2)	2.8 (3)
Psychotic spectrum disorder	10.2 (11)	0.9 (1)
Obsessive compulsive disorder	13.9 (15)	0.0 (0)
Bipolar and related disorders	7.4 (8)	0.0 (0)
Oppositional defiant disorder	0.9 (1)	0.0 (0)
Unspecified personality disorder	0.9 (1)	0.0 (0)
Borderline personality disorder	7.4 (8)	0.0 (0)
Narcissistic personality disorder	0.9 (1)	0.0 (0)
Gender dysphoria	1.8 (2)	0.0 (0)
Attention deficit hyperactivity disorder	20.3 (22)	0.0 (0)
Autism spectrum	15.7 (17)	0.0 (0)
Intellectual disability	7.4 (8)	0.0 (0)
Tourette’s syndrome	0.9 (1)	0.0 (0)
Learning disorder	0.9 (1)	0.0 (0)
Paraphilic disorder	2.8 (3)	51.8 (56)
Pedophilic disorder	––	35.2 (38)
Other specified – Hebephilia	––	27.8 (30)
Voyeuristic disorder	––	0.9 (1)
Exhibitionistic disorder	––	1.8 (2)
Frotteuristic disorder	––	0.9 (1)
Sadistic disorder	––	1.8 (2)
Transvestic disorder	––	0.9 (1)
Autogynephilia	––	1.8 (2)
Zoophilia	––	0.9 (1)

*Note.* Past diagnoses were based on client self-report, whereas current TFC diagnoses were based on the results of the TFC diagnostic assessment.

Among the 108 clients who completed the diagnostic assessment, 55.6% (*n* = 60) received a current mental health disorder diagnosis at the time of the assessment (inclusive of paraphilic disorders; see second column of Table 2). For paraphilic disorders specifically, 51.8% (*n* = 56) of TFC clients qualified for a paraphilic disorder diagnosis following the TFC diagnostic assessment with 35.2% (*n* = 38) receiving a diagnosis of Pedophilic Disorder and 27.8% (*n* = 30) receiving a diagnosis of Other Specified Paraphilic Disorder – Hebephilia. Further, 20.4% (*n* = 22) received multiple paraphilic disorder diagnoses.

Table 3 provides descriptive statistics for self-report measures of mental health symptoms and functional impairment gathered as part of the TFC diagnostic assessment (*n* ranged from 92 to 97 due to missing data). TFC clients average score on the WHODAS was 24.0, which falls at the 85th percentile compared to the normative sample. Almost half of the sample (45.3%, *n* = 44) reported at least mild impairment due to a physical or mental health concern. The results also suggest significant depressive and anxiety symptomatology in TFC clients, with 64.1% (*n* = 59) and 58.9% (*n* = 56) of clients experiencing at least moderate levels of depressive and anxiety symptoms, respectively. Alcohol and drug misuse were less notable in the sample, with 12.9% (*n* = 12) of clients reporting alcohol use within the hazardous or alcohol dependence ranges and 13.0% (*n* = 12) of clients reporting substance use within the intermediate and substantial severity ranges.

**Table 3.** Clinical Characteristics of Talking for Change Clients Who Participated in a Diagnostic Assessment and Completed Clinic Mandated Self-Report Questionnaires

​	*M* (*SD*) [Range]	% (*n*)
Functional impairment (WHODAS 2.0; *n* = 97)	24.0 (10.1) [12–54]	
No impairment		54.6 (53)
Mild impairment		27.8 (27)
Moderate impairment		16.5 (16)
Severe impairment		1.0 (1)
Extreme impairment		0.0 (0)
Hypersexuality (CBSD-DI; *n* = 93)	11.0 (4.4) [0–18]	
Depressive symptoms (PHQ-9; *n* = 92)	12.7 (7.1) [0–27]	
None to minimal		16.3 (15)
Mild		19.6 (18)
Moderate		22.8 (21)
Moderately severe		22.8 (21)
Severe		18.5 (17)
Anxiety (GAD-7; *n* = 95)	10.4 (5.8) [0–21]	
Minimal anxiety		20.0 (19)
Mild anxiety		21.1 (20)
Moderate anxiety		33.7 (32)
Severe anxiety		25.3 (24)
Alcohol use (AUDIT; *n* = 93)	3.6 (5.5) [0–39]	
Absence of alcohol-related problems		21.5 (20)
Low-risk consumption		65.6 (61)
Hazardous consumption		8.6 (8)
Possible alcohol dependence		4.3 (4)
Drugs use (DAST-20; *n* = 92)	2.2 (3.2) [0–15]	
No drug use		42.4 (39)
Low severity		44.6 (41)
Intermediate severity		8.7 (8)
Substantial severity		4.3 (4)
Severe use		0.0 (0)

*Note.* 108 individuals were offered a TFC assessment and were expected to complete a standard battery of questionnaires; however, there is missing data for several participants who did not complete questionnaires or missed certain questions (sample size varied from 92-97 across measures).

WHODAS 2.0 = World Health Organization Disability Assessment Schedule 2.0; CBSD-DI = Compulsive Sexual Behavior Disorder – Diagnostic Inventory; PHQ-9 = Patient Health Questionnaire; GAD-7 = Generalized Anxiety Disorder −7; AUDIT = Alcohol Use Disorders Identification Test; DAST-20 - Drug Abuse Screening Test.

Table 4 provides descriptive statistics for mental health treatment, suicidality, adverse childhood experiences, and risk and protective factor profiles for those who participated in the extended evaluation (*n* = 40). Just under two-thirds of this subset of TFC clients had undergone mental health treatment. Most of the evaluation clients reported a history of suicidal ideation (77.5%, *n* = 31), with 40% (*n* = 16) reporting current suicidal ideation and 7.5% (*n* = 3) reporting a past suicide attempt. Just under half the sample endorsed experiencing any form of abuse in childhood, with the most frequent being physical, emotional, and sexual abuse. Most evaluation clients self-reported a paraphilic interest (97.5%, *n* = 39), which was a broader variable than diagnosis because it was determined to be present if the client endorsed relevant sexual interest (i.e., distress and/or impairment not required for the interest to be present). Of the different interests, pedohebephilia was the most frequently endorsed paraphilia (80.0%, *n* = 32) and sexual interest in exhibitionism, non-consenting partners, and sadomasochism being relatively frequent. For the 31 evaluation clients who had information about exclusivity of self-reported pedohebephilic interests, 32.3% (*n* = 10) experienced non-preferential pedohebephilic interests, 29.0% (*n* = 9) experienced preferential pedohebephilic interests, and for 38.7% (*n* = 12) preferentiality was unknown.

**Table 4.** Dynamic Risk Factors, Protective Factors, Offense History, and Clinical Features of Talking for Change Clients Participating in the Evaluation (*n* = 40)

​	% (*n*)
Clinical features	
Mental health treatment	
Past mental health treatment	62.5 (25)
Current mental health treatment	30.0 (12)
Current psychotropic medication	57.5 (23)
Past sexuality-specific treatment	35.0 (14)
Suicidality	
Past suicidal ideation	77.5 (31)
Current suicidal ideation	40.0 (16)
History of suicide attempts	7.5 (3)
Adverse childhood experiences	
Any abuse	42.5 (17)
Emotional abuse	15.0 (6)
Physical abuse	17.5 (7)
Sexual abuse	12.5 (5)
Neglect	5.0 (2)
Witnessed domestic violence	0.0 (0)
Other forms of abuse/adversity	20.0 (8)
Paraphilic interests	97.5 (39)
Pedohebephilia	80.0 (32)
Exhibitionism	17.5 (7)
Voyeurism	30.0 (12)
Frotteurism	10.0 (4)
Fetishism	22.5 (9)
Non-consent	30.0 (12)
Transvestic fetishism	10.0 (4)
Zoophilia	17.5 (7)
Uro/coprophilia	12.5 (5)
Sadism/masochism	25.0 (10)

Risk and protective factors	​	​
​	*M* (*SD*) [range]	Factor present % (*n*)
VRS:SO – Dynamic risk factors^1^		
Total score	17.5 (4.2) [10–25]	
F1: Sexual deviance	8.8 (2.2) [4–14]	
F2: Criminality	3.4 (2.2) [0–9]	
F3: Treatment responsivity	3.1 (2.0) [0–6]	
Sexually deviant lifestyle	1.9 (0.6)	84.6 (33)
Sexual compulsivity	1.7 (1.0)	61.5 (24)
Offense planning	1.2 (1.0)	43.6 (17)
Criminal personality	0.2 (0.4)	2.6 (1)
Cognitive distortions	0.7 (1.0)	23.1 (9)
Interpersonal aggression	0.8 (0.8)	23.1 (9)
Emotional control	1.1 (1.1)	35.9 (14)
Insight	1.2 (0.9)	43.6 (17)
Substance abuse	0.6 (1.0)	15.4 (6)
Community support	1.0 (0.8)	35.9 (14)
Release to high-risk situations	1.3 (1.0)	38.4 (15)
Sex offense cycle	1.6 (0.8)	56.4 (22)
Impulsivity	0.7 (1.0)	25.6 (10)
Compliance with supervision	-	-
Treatment compliance	0.2 (0.4)	0.0 (0)
Deviant sexual preference	2.3 (0.5)	97.4 (38)
Intimacy deficits	1.1 (1.7)	35.9 (14)
SAPROF-SO^1^		
Total score	29.2 (9.6) [7–46]	
F1: Resilience	5.4 (1.7) [1–8]	
F2: Adaptive sexuality	2.3 (2.0) [0–6]	
F3: Prosocial connection & reward	4.9 (2.4) [0–8]	
Adaptive schema	2.1 (1.1)	71.8 (28)
Empathy	3.2 (0.9)	94.9 (37)
Coping	1.7 (1.1)	61.5 (24)
Self-control	2.8 (1.1)	87.2 (34)
Attitudes toward rules and regulations	3.3 (1.1)	89.7 (35)
Sexual self-regulation	1.1 (1.0)	33.3 (13)
Prosocial sexual interests	1.5 (0.6)	53.8 (21)
Prosocial sexual identity	1.3 (1.4)	43.6 (17)
Intimate relationships	1.2 (1.4)	35.9 (14)
Goal directed living	2.1 (1.4)	64.1 (25)
Work	2.5 (1.6)	71.8 (28)
Leisure activity	1.1 (1.4)	33.3 (13)
Social network	2.4 (1.1)	82.0 (32)
Emotional connection to adults	2.8 (1.2)	69.2 (27)

*Note.* The clinical features data were extracted from the diagnostic assessment report using a coding form. The risk and protective factors were collected from the pre-treatment interview administered by the research analyst. The VRS:SO items were deemed present if they were scored either a 2 or 3 (indicative of a treatment need). For the SAPROF-SO, anything scored 2 to 4 was deemed present, which represents that the problem was present to some extent or was present. VRS: SO = Violence Risk Scale – Revised; SAPROF-SO = Structured Assessment of Protective Factors against Sexual Offending, version 1 *n* = 39.

Few evaluation clients had a history of detected sexual offenses; however, 67.5% (*n* = 27) reported purposeful attempts to find and/or access CSEM (inclusive of both detected and undetected offending, as well as real and/or fictional CSEM). In relation to contact offending, it was found that 10.0% (*n* = 4) reported past contact sexual offending (against children) that was undetected by the criminal justice system. When examining the risk factors related to sexual offending—as assessed by the VRS:SO—the total score for the sample (*M* = 17.5 of a possible score of 51, *SD* = 4.2) suggesting that most factors were not identified as treatment needs for clients. The clients in this sample tended to most frequently experience problems in the sexual deviance domain relating to risk-relevant sexual preferences, problems in sexual self-regulation, and risk-relevant sexuality characterizing one’s lifestyle (see Table 4). Lack of community supports (35.9%, *n* = 14), intimacy deficits (35.9%, *n* = 14), and lack of insight into one’s own risk-relevant problems (43.6%, *n* = 17) were relatively frequent problems for TFC clients. Dynamic risk factors relating to general criminality were infrequently identified as problematic for TFC evaluation clients (e.g., criminal personality present for 2.6% of clients, substance abuse present for 15.4% of clients).

Additionally, there were several protective factors that were frequently present for TFC clients. The average score in the protective domain of resilience was highest, followed by prosocial connection and reward, and adaptive sexuality. In terms of individual protective factors, the more prevalent protective factors included empathy, attitudes toward rules and regulations, self-control, emotional connection to adults, and adaptive schemas (see Table 4).

### R03: Client Satisfaction With the TFC

RO3 involved a thematic analysis of program satisfaction interviews. The results are presented across five themes and illustrative quotes are provided.

#### “I Couldn’t Imagine What the Conversation Would Look Like”: Overcoming Fear, Discomfort, and Stigma

The central component of this theme focused on the significant challenges clients overcame to engage with the program. For some, this was closely related to their knowledge about negative public attitudes toward those with sexual interest in children. One client described this succinctly when he stated “imagining everybody hates you because of that, the whole, the whole system hates you. The legalities hate you, you know what I mean? Like, the whole world kinda hates you” (Client #5). These negative perceptions translated into internal barriers, as clients reported a range of emotions at the outset of the program including fear, anxiety, general discomfort, or mistrust. For example, one client described the experience of completing the online self-referral form noting that there was “possibly a bit of paranoia, a little bit of fear with respect to, ah, what is this, this, this government agency [in reference to the hospital] that I’m, filling out a form for…who is on the receiving end of this*”* (Client #4). Overall, concerns focused on the virtual format of the program, documentation on their medical chart, and wider socio-legal concerns. These concerns were balanced with their understanding of the purpose of the program and knowing that OCSEA perpetration prevention services are hard to find. To overcome barriers, clients suggested it may be helpful to have introductory materials following referral letting them know more about the program, informing them that everyone else in group is in a similar situation, and acknowledging the significant step they have taken in completing a referral to the program.

#### “A Weight Has Been Lifted”: The Power of Acceptance and Accountability

The second theme focused on the ways in which other clients and clinicians facilitated feelings of acceptance (inclusive of self-acceptance and acceptance from others), connection to others, and accountability regarding their behavior. Clients also discussed an internal process of acceptance related to understanding their sexuality and incorporating it into their identity:

It’s a kind of liberation that occurs. It’s just another, it’s another layer of coming out…. as frightening as it is to, to take on that, that identity, that label of pedophile or MAP [Minor Attracted Person] or whatever you want to say, once it is there and once it's, once it's, once you achieve a certain level of comfort with that self-identity, there is a, there is a kind of weight that lifts in terms of the, the honesty of living, [inaudible word] and with integrity, ah with that fact…. uh, able to just, uh, walk through the world, uh, because it’s no longer, uh, a secret. (Client #4)

The component of self-acceptance was concretely linked to later stages in the program where clients learned explicit strategies to address sexual based concerns. As mentioned by one client, acceptance helped them apply other skills to their daily functioning, “that was probably the most meaning, because without that, I don’t think I could have, like, really, mm, like, felt like I could like, apply any of the tools*”* (Client #10).

Clients highlighted the importance of accountability for their behavior, which is nicely illustrated by a quote describing group dynamics, “We definitely bounced off each other’s ideas or it was a great idea to kind of be in a group in this type of particular situation because it kind of just brought out, like… red flags that people knew, but weren’t addressing*”* (Client #11). Some expressed concern about loss of accountability at the end of the program and wondered about the value of a follow-up program. Further, clients highlighted that facilitators helped convey acceptance and accountability. For example, one group member described a “turning point” in therapy where they were engaged in risky online behavior and were directly challenged by facilitators in a supportive and helpful manner.

#### “Feeling Authentically Connected to the Others”: The Value of Group

A related theme focused on the way the program provided a means of connection with other clients, which was pivotal because it allowed them to see themselves and learn from experiences of others in similar situations. For example:

They shared, um, a lot of positive, um, insights, I remember one group member saying ‘Um, it’s never easy, but it does get easier.’ I remember, um, another group member actually experienced what I had experienced. That was very helpful. Just to see him, you know, alive and there. (Client #3)

A key aspect of the program appeared to be that client could imagine the experiences of other group members happening to them. As one group member stated, “hearing someone firsthand, explain kind of what the mismanagement of their attraction like led to is really scary to me, because I couldn’t picture myself in that situation up until that point” (Client #9). Another client shared how hearing another client’s experience of disclosing his sexual interest to a family member helped them to challenge their own negative preconceptions about such disclosures. Some expressed a preference for these kinds of exploratory group process, for example, one group member highlighted that they wanted more group discussion about the overall experience of living with a sexual interest in children. An informal support component of the program was also highlighted by some with one client suggesting a pre-session virtual get together where members could just talk with one another without heavy emphasis on skills.

#### “It Is So Orderly and Clinical”: Challenges With Mental Health-Based Programs and a One-Size Fits All Approach

The fourth theme highlighted some of the challenges that can occur in the delivery of group-based programs, especially logistical challenges that can occur in hospital or virtual settings. In discussing this, one of the clients shared their experience completing the early stages of the program, including completing the online intake form. They remarked that it felt like they were talking to a computer screen and recognized a tension in that it was a necessary step to assess their fit for the program, but it also felt “a little less than therapeutic at times because it is so orderly and clinical” (Client #4). Further, other clients expressed apprehension about the virtual format and a desire for more individualized support.

A challenge in mental health programming is that there is often a greater demand for services, which translates into increased wait times. Some clients commented on difficulties associated with the wait time to access services, “not everybody is willing to stay in line, uh, and these are… emotional times. There are times that you hate yourself so much that it can be dangerous to, or you can be dangerous to yourself” (Client #5). In addition to logistical challenges of group-based mental health treatment, some clients provided feedback about the linkage between content learned in group and relevant outcomes. There was a sense some of the content—especially related to general mental health skills—could miss the needs of some but address the needs of others and some of the general skills were not clearly linked with OCSEA perpetration prevention. One member described this challenge rather succinctly via an example of having an urge to use CSEM:

Now, stop, and now that’s the exact point when mindfulness and thought records and all this stuff that we we’re learning, that’s exactly when it’s supposed to, to work. That’s, not- some, and so I felt that if we’re going to learn all this stuff, let’s learn it in the context of why we’re learning it and so typing in those first couple letters gets you, starts to get you in that situation mentally that you can now apply all this stuff. (Client #8)

#### “There Is a Sense of Agency, That It Is Not Just One-Sided Delivery” – The Value (and Challenges) of Evaluation

Clients recognized that establishing the effectiveness of the program through program evaluation was crucial. Despite this, clients identified some difficulties with participating in such research. Despite discomfort with some of the questions that were asked of them, clients saw the value of participating in a relatively intensive evaluation as illustrated in the below statements:

Some of the questionnaires, were a little bit jarring at first, um, but obviously it’s information that definitely needs to be required to kind of give a sense of, like, what type of direction, like this, I have- this attraction is, um, so obviously I was kind of taken a back first, um, but I kind of realized, like, okay, well, mainly this is for, this is the benefit for, like, like for both parties basically, so, um, during that, like whole questionnaire and stuff like that, like, the one I did by myself was, like, I, I was able to get through it pretty well as well as, like, um, the assessment. (Client #11)

Interestingly, some clients seemed particularly interested in the results of the evaluation and how their responses might change over time. One group member remarked that they often reflected on changes they experienced throughout the program, which was prompted by the weekly questionnaires. In contrast, group members raised criticism relating to the length of the self-report measure battery and the validity of the measures.

## Discussion

The feasibility study results are relatively positive in that 162 adults were referred in the first three years of implementing the TFC perpetration prevention program. Referral rates increased over time suggesting increased demand for services. Taken as a whole, referral rates indicate that there is a need for these clinical services, and it is feasible to reach this client population in a mandatory reporting jurisdiction.

From a feasibility perspective, a logical and more nuanced question concerns the characteristics of clients are who seek services at TFC, with the most critical being whether they are true secondary prevention clients. TFC was intended to provide preventative therapy to individuals with sexual interest in children or those who are at-risk of OCSEA perpetration. TFC clients reported low rates of offending histories that were known to legal authorities, which also extended to undetected contact sexual offending against children; however, these estimates should be viewed as conservative estimates given that they relied exclusively on self-report and individuals may have been concerned about mandatory reporting obligations (see below). From a contact offending standpoint, it appears that TFC does reach individuals prior to offending, which is relatively promising. Nonetheless, just over two-thirds of TFC clients reported purposeful attempts to access CSEM, which in Canada is inclusive of material depicting real children as well as fictional material (e.g., drawings, AI generated material, written text). The significant use of CSEM in our sample is similar to that observed in other perpetration prevention programs (e.g., Beier et al., 2015) and suggests that we should think more critically about how we reach people prior to CSEM offending.

Another relevant consideration is that the target group for TFC includes individuals without sexual interest in children who were concerned about their risk of offending. Although most individuals in the evaluation (*n* = 40) endorsed some degree of sexual interest in children, 20% of evaluation clients reported no such interest. The number of individuals was too small to provide meaningful analyses on their risk profiles; however, a closer look at these individuals found that five of them (62.5%) had engaged in CSEM and/or other forms of offending (e.g., non-contact sexual offending). Thus, it appears that TFC also attracts individuals without sexual interest in children, many of whom had already crossed illegal boundaries, which may have prompted them to seek services. As TFC continues and the sample size increases, it will be useful to conduct a more direct comparison between individuals with and without sexual interest in children.

### Clinical Profiles of Clients in the TFC Program

The pattern of dynamic risk and protective factors found for TFC client sample presents an interesting clinical picture. This client population presents with problems relating to risk-relevant sexuality, sexual self-regulation, and risky behavior, along with difficulties in their interpersonal and intimate relationships. Further, qualitative analyses suggested that clients appreciated the ability to receive services that promoted acceptance of their underlying sexual interests while also promoting behavioral accountability for their choices. A counterbalance to this potentially risky combination of problems, is that TFC clients tended not to display problems with antisociality, impulsivity, and cognitive distortions, which are psychological processes that might potentiate these existing problems relating to sexuality. Further, TFC clients were frequently rated as having several protective factors including, empathy for others, adaptive coping strategies, self-regulation of emotions and behavior, and goal-directed lifestyles. This pattern of risk and protective factors may not be entirely surprising for a client population that is voluntarily seeking support around their sexual behavior to ensure they do not offend and harm others. This pattern of results provides evidence that TFC is reaching a client population that has risk-relevant needs and provides clear empirical direction for perpetration prevention programs in terms of risk factors to address and the kinds of protective factors to leverage in treatment with these clients.

The mental health profile of TFC clients is noteworthy as clients enter treatment with a high mental health burden, most significantly relating to depressive and anxiety symptomatology and suicidality. TFC clients also tended to report childhoods that were marked by abuse and other forms of adversity. This pattern of elevated internalizing psychopathology, elevated suicidality, elevated adverse childhood experiences, lower substance use problems, and lower personality psychopathology is consistent with past research with relevant samples (de Tribolet-Hardy et al., 2025; McPhail, 2024).

Another notable pattern in the mental health profile of these clients was that few reported past paraphilic diagnoses (2.8%) while over half of TFC clients were diagnosed with paraphilic disorders during the diagnostic assessment. This specific finding suggests that contact with TFC is likely the first time most clients have sought mental health support for sexuality-related reasons or disclosed atypical sexual interests to health professionals, though many of them have sought previous mental health treatment. The qualitative interviews also highlighted the difficulties that clients faced accessing services in the past and their significant concerns about engaging in services. Relatedly, these clients had concerns about disclosing their sexual interests to their personal support network as well. These findings are in accordance with past research that highlights barriers to treatment in this client population and that clients often struggle to find personal or professional support (e.g., Chronos et al., 2024).

The risk and protective factor profiles and the mental health needs of our clients raise a potential puzzle from a feasibility perspective: Can perpetration prevention programs feasibly and effectively address all the needs these clients present with? Given that clients often report a pattern of both risk-relevant problems and mental health needs, are some clients better served by general mental health interventions and if so, how can we best identify these clients? In the case of TFC, the program currently provides 32 hours of treatment over 16 weeks (and an additional two sessions of individual therapy), with most of the program geared toward sexuality- and risk-specific issues. This is a significant amount of clinical service, and it remains to be seen if participating in risk-specific intervention to prevent OCSEA perpetration has a notable impact on client mental health. Importantly, a high percentage of clients who participated in the evaluation (*n* = 40) reported additional mental health treatment (i.e., psychotropic medication or general mental health treatment). Nonetheless, this introduces a challenge that was uncovered via qualitative results as some clients have a more sophisticated understanding of general clinical skills for mental health problems and really want to focus on their application to risk-relevant domains, whereas others require considerable support learning this information outside of the context of risk as it was their first exposure to mental health services.

Questions of feasibility are also relevant to assessment practices. In tertiary OCSEA prevention programs within the criminal justice system, it is routine to use clinician-rated measures to assess for dynamic risk factors, because the presence of these factors informs treatment goals for individual clients. It is important to draw out the fact that the present data are novel: this is the first time data from interview-and file review-based, observer-rated measures of dynamic risk and protective factors have been provided for individuals who voluntarily seek treatment within secondary OCSEA prevention programs. These measures have been adapted in the TFC program for a new client population, which represents an off-label use of the VRS:SO and SAPROF-SO. The use of these measures was, to a certain degree, feasible. These measures were able to be rated for TFC clients, clients tolerated both this component of the evaluation, and doing so was feasible from a staffing standpoint; however, during the feasibility evaluation it was clear that certain items were not relevant or rateable for TFC clients (e.g., the Compliance with Supervision item on the VRS:SO). The focus of these measures on assessing whether factors were associated with *past* OCSEA perpetration was clearly not applicable for a substantial portion of TFC clients and this scoring focus needed to be adapted in this context.

### Implications

There are several implications for non-anonymous OCSEA perpetration prevention programs. Most clients did not have a self-reported history of detected or undetected contact offending, but many accessed CSEM. Readers are reminded that rates of contact offending especially are likely conservative estimates given client concerns about mandatory reporting (see below discussion). Despite the importance of providing services to those with histories of undetected sexual offending, these results suggest that we may be reaching some people later than we initially hoped (i.e., prior to any form of illegal sexual behavior, including illegal online behavior).

There are several possibilities for this trend. First, it is possible that the program advertisements (with a heavy emphasis on OCSEA prevention) appeal most to those with a history of undetected sexual offending. Alternatively, it may be the case that people simply do not know what situations are risky or what risk-relevant problems are present in their lives and they do not detect when to seek help before accessing CSEM online (raising an important question on how to best reach people who are on a path that could result in CSEM use). It is possible that a different approach to advertising that emphasizes mental health needs could impact the types of individuals who seek out OCSEA perpetration prevention programs, as this framing is often consistent with client priorities (Lievesley et al., 2023). A second possibility is that there is a high rate of undetected offending, mostly CSEM, among those with sexual interest in children, which should be the focus of future research.

A relevant consideration in a mandatory reporting context is that clinicians must balance client confidentiality and mandatory reporting obligations. Mandatory reporting is a complex issue and each jurisdiction in Canada varies slightly in the conditions that must be met before there is a duty to report when an identifiable child is being abused or is at-risk of being abused (though not all jurisdictions include risk of being abused as reportable; see McPhail et al., 2018 for a discussion of these issues). Most TFC clients had no self-reported history of contact offending. In instances when clients disclosed past offending, clinicians attempted to gather more information to determine if reporting was required. In most cases, a report was not necessary because the offenses were historical (e.g., the child who was harmed is now an adult) and there was no ongoing risk to children, the offense was previously investigated, or there was insufficient information to file a report. Further, some provinces require reporting when one directly encounters CSEM, but this applies to discovering material rather than to clients’ past use, so client disclosures of CSEM use typically do not trigger a report. Nonetheless, navigating these issues in a mandatory reporting context within a perpetration prevention program is complex and merits further study as it is a clear challenge that clinicians must navigate.

A related issue is that not all individuals endorsed a sexual interest in children or received a diagnosis consistent with this interest. As previously discussed, OCSEA is a multifactorial phenomenon that is driven by a wide range of risk factors (e.g., Seto, 2018). OCSEA perpetration prevention programs, including TFC, must carefully think about the best way to reach individuals who are concerned about their risk or behavior for a range of issues (Stephens et al., 2022). Despite the importance of this, the potential diversity in client profiles creates additional challenges for program delivery. During program satisfaction interviews, clients noted the challenges of group-based programs that often take a one-size-fits-all approach. It is possible that the needs within this client group could be different and certainly some of our program content (e.g., acceptance of sexual interest in children, management of sexual fantasies) would be less applicable to those without such an interest. Relatedly, some clients with more involvement with the mental health system highlighted that the TFC content often included skills learned in other programs. Nonetheless, clients stressed the value of group-based programs in that they allow them to connect with others in similar circumstances and promote acceptance and accountability, so this appears to be an important ingredient to the program.

Overall, it is worth noting that perpetration prevention programs could recruit a heterogeneous client population that differs in important ways. As previously mentioned, 20% of our sample did not have pedohebephilic interest, despite many previous perpetration prevention programs recruiting just this group (e.g., Beier et al., 2009, 2015). Another key difference was that not everyone in the sample had offended. The way that these different client populations interact with the program and its content may differ and impact their satisfaction with the program. For example, fear of discovery might be less salient for individuals with no history of offending. The issue of different client profiles and how they interact and/or benefit from perpetration prevention programming should be explored in future studies.

Lastly, it is important to highlight that TFC clients had significant mental health needs as highlighted by the rate of past and current diagnoses as well as their pre-treatment scores on measures of anxiety, depression, and alcohol misuse. The diagnostic complexity of this population is consistent with the wider literature that highlights the significant mental health needs of individuals with sexual interests in children (e.g., Chronos et al., 2024; Levenson & Grady, 2019; Lievesley et al., 2023) and a Swedish study of a perpetration prevention program that found a high rate of mental health comorbidity. Like our findings, Affective Disorders were the most common comorbidity, though they also had a high incidence of Personality Disorders (de Tribolet-Hardy et al., 2025). Perpetration prevention programs must carefully consider diagnostic complexity and comorbidity in the delivery of their programs, including the way they will monitor mental health and suicide risk and the types of adjunctive services that may be required.

### Limitations

The present feasibility study should be interpreted with several limitations in mind. First, our sample size was relatively small, though this is less of a concern for feasibility studies. For several clinical variables and the dynamic risk and protective factors (see Table 4) there was only complete information for evaluation clients (*n* = 40). There was also a significant proportion of the program data that was unknown. Further, the results cover the first three years of the program, and there has been a significant uptake in referrals. It is possible that with a wider reach, continued improvements in marketing, and longer implementation times that client characteristics might shift over time. Relatedly, we did not adequately track the number of clients that refused the additional evaluation, which precluded additional analyses on differences between those who refused the additional evaluation procedures. Further, the results for program satisfaction only included individuals who completed the program with one exception. Although our overall retention was strong, it would have been helpful to have data from these participants on their experience in the program. Further, we did not engage in a process of member checking to ensure our interpretation of material from the program satisfaction interview fit the experience of participants, though the appropriateness of this approach is debated (Braun & Clarke, 2013).

### Conclusions and Future Research Direction

A main conclusion from this feasibility evaluation is that the implementation of OCSEA perpetration prevention interventions is feasible in a Canadian context where mandatory reporting laws exist. We believe the results are promising and suggest that TFC has reached its intended client population and shows year-on-year increases in the number of individuals seeking support. These individuals present with a range of clinical needs and risk factors for OCSEA perpetration, raising the need for such programs to offer comprehensive mental health assessment to best serve this client population. Clients tended to tolerate TFC group psychotherapy, seeing value in the program for their specific circumstances, while also noting concerns and difficulties with the program.

At present, there is a need for more rigorous evaluations of OCSEA perpetration prevention programs like TFC. To date, the research has been relatively limited (Seto et al., 2024; Stephens et al., 2022) and there is only one study that used a randomized controlled trial to examine the overall effects of an anonymous self-directed therapy program (Lätth et al., 2022). There is also a need for further evaluations of youth focused perpetration prevention programs. More rigorous evaluations are needed, and innovative methodologies are crucial so that they can examine the ultimate outcome of OCSEA perpetration. Recent developments in the field suggest that more rigorous evaluations will start to appear in the literature to address the overall effectiveness of this approach, including an upcoming randomized controlled trial of the TFC therapy program. Future research could also examine the way that different client profiles influence reaction to program content and its efficacy.

## Supplemental Material

Supplemental Material - A Feasibility Study of a Canadian Child Sexual Abuse Perpetration Prevention Program Supplemental Material for A Feasibility Study of a Canadian Child Sexual Abuse Perpetration Prevention Program by Skye Stephens, Ian V. McPhail, Ainslie Heasman, Cory Gerritsen, Sarah Moss, Stéphanie Chouinard-Theiverge in Sexual Abuse

## Notes

1. We acknowledge that secondary prevention can be defined differently depending on the source that is being used for the definitions. For example, in the United States the Centre for Disease Control would include targeting of at-risk populations as primary prevention.

2. All clients were expected to complete clinic administered questionnaires; however, due to missing data, the n’s for these various measures range from 92 to 97.

3. Clients who participated in the additional evaluation procedures also completed the following: an interview with the research analyst following treatment; a battery of self-report measures of risk factors and mental health concerns at pre- and posttreatment; and self-report measures of mental health, acute risk, and the therapeutic relationship each week for the duration of their involvement in treatment. Data from these other measurements and time points will be present in other manuscripts focusing on issues beyond the feasibility of the TFC program.
