# Supplementary material for: A Feasibility Study of a Canadian Child Sexual Abuse Perpetration Prevention Program
Source: Sex Abuse. 2026 Jun 18;38(7):849–83. doi: 10.1177/10790632261460836 (PMC13534775; doi:10.1177/10790632261460836)
Supplement: Supplementary file 1 [file sj-pdf-1-sax-10.1177_10790632261460836.pdf]

<u>Online Supplementary Material</u>

We have included a copy of the program satisfaction interview. We have included the general information shared with the client, but excluded the instructions included for the interviewer. The interview was a semi-structured interview so the interviewer was allowed to deviate from the interview guide where necessary and to further explore important topics.

<u>Interview Guide</u>

Information shared with clients The first thing to mention is the purpose of the interview today, which is to get your perspective on the prevention program you participated in, regardless of whether you completed the program. We would like to learn about your experience from the time you were referred to the time you completed the program with the post-treatment assessment. Even if you did not complete all elements of the program or the evaluation we would still like to learn about your experience. The aim of the interview is to improve the program and its evaluation in future years. The interview should take approximately 60 minutes.

Review informed consent information.

Do you have any questions before we begin?

Note to interviewer: The interview guide is designed to structure the interview in some ways but to leave it as open ended as possible so that you can follow the lead of the interviewee. The aim of the interview is to cover the main topics as reflected by the questions, but to do our best to allow the interviewee to guide the interview. The interview is sectioned by theme, and you can feel free to follow the lead of the interviewee within each of these themes. You will want to have a good memory of the interview guide so that you can ask questions that we are hoping to have addressed by the interviewee that they may not have brought up by themselves. Given that the interview is semi-structured, do not be afraid to explore responses that deviate from the interview guide. Any information the interviewee provides about prevention program is useful and it is possible there are issues we have not considered in the interview guide that are important to explore with interviewees.

There are probes that are included to elicit further information from interviewees about their reasoning or to help clarify their response. If you are unclear about an answer or want the interviewee to say more, feel free to use general prompts to follow-up. Some useful example prompts might be: "Can you tell me more about XXXXX"; "Can you take me through XXXX in a bit more detail." We also will want clients to expand on the reasons for their recommendations, so please query why they think different issues they bring up are important.

You can use other prompts and follow-up as necessary but just remember to try and ask open-ended questions that are <u>not</u> leading (e.g., why do you think the program is the

best thing ever; tell me all the ways this program radically changed your life for the better). We also want to try and ensure the interview takes approximately 60 minutes, so it is also important to keep the interview focused and moving at an appropriate pace.

Do you consent to participate in the interview?

If yes: Thank you for agreeing to participate. I have started the audio recording.

If no: Okay thank you for your consideration.

<u>Reaction to Program Content</u>

We would like to start by getting your reaction to the content of the prevention program.

Before we do this can you please let us know if you completed the following study components ***[Note to interviewer: You may need to explain the component in a bit of detail if clients are not sure what you are referring to]:***

- *Self-referral to the program:* y/n
- *Diagnostic assessment:* y/n
- *Pre-treatment assessment:* y/n
- *Prevention Group:* y/n
    - **Probe:** # of sessions reported, if dropped out when did this happen
    - **<u>Follow-up question:</u>** If answer is no to prevention group ask them if they completed individual prevention treatment
        - **Probe:** # of sessions reported, if dropped out when did this happen
- *Weekly session surveys:* y/n
    - **Probe:** # of weekly session reports y/n
- *Post-treatment assessment:* y/n

Please tell me about your overall experience in the group/individual therapy.

- **Probe:** What was it like to be a group member.
- **Probe:** Would you recommend this group to a family or friend if they needed it? Why or why not?

- **<u>Follow-up question if not addressed:</u>** What was the most valuable part of the group?

- **<u>Follow-up question if not addressed:</u>** What was the least valuable part of the group?

Tell me about a moment in group/individual therapy that was transformative for you

- **Probe:** Can also ask about the most meaningful moments in therapy if client has trouble generating.

<u>Referral and Assessment Process</u>

We next want to ask some more questions about the referral process and your experience participating in the various assessments (both interviews and online surveys).

Tell me about your experience being referred to the program

- **<u>Follow-up question if not addressed:</u>** Were there any barriers to the referral process?

- **<u>Follow-up question if not addressed:</u>** Is there anything that can be improved in the future to make the referral process easier for clients?

- **<u>Follow-up question if not addressed</u>**: Is there anything that would have been useful to know about before your referral to the program?

What were your initial reactions when you discussed the informed consent for the group and the study?

- **Probe:** Remind them of the consent process and different forms they signed and ask for feedback on each of them or get collective feedback. [***Note to interviewer: It is important to clarify what informed consent form they are discussing if it appears to be very specific to one particular form and/or process***]

- **<u>Follow-up question if not addressed:</u>** Is there anything we can do to improve the informed consent process?

- **<u>Follow-up questions if not addressed:</u>** Do you have any suggested changes for the informed consent form?

Tell me about your experience of the diagnostic assessment?

- **<u>Follow-up question if not addressed:</u>** What sorts of things were covered in the diagnostic assessment that you believed were very important

- **Follow-up question if not addressed:** What sorts of things were not covered in the diagnostic assessment that you think would have been important to cover

- **Follow-up question if not addressed:** Any suggested changes for the diagnostic assessment

What was your experience of the pre and post-treatment assessment?

- **Follow-up question if not addressed:** What sorts of things were covered in the pre and post treatment that you believed were very important

- **Follow-up question if not addressed:** What sorts of things were not covered in the pre and post treatment that you think would have been important to cover

- **Follow-up question if not addressed:** Any suggested changes for the pre and post treatment assessment

What was your experience like completing the weekly session surveys?

- **Follow-up question if not addressed:** What sorts of things were covered in the weekly surveys that you believed were very important

- **Follow-up question if not addressed:** What sorts of things were not covered in the weekly surveys that you think would have been important to cover

- **Follow-up question if not addressed:** Any suggested changes for the weekly survey assessment

Interview Close

Note to interviewer: The remainder of the questions will bring the interview to a close. It is okay to ask the interviewee for a second to review your notes/interview questions to ensure that you have addressed the interview questions. Do not shy away from doing this because we want to ensure we have addressed the main areas in the interview guide and adequately followed-up on information the interviewee provided.

Before we wrap up, is there anything that we did not cover during our time today that you think is important to add?
